# Innominate artery reconstruction during emergent surgery for acute aortic dissection

**DOI:** 10.1186/1749-8090-8-S1-O70

**Published:** 2013-09-11

**Authors:** S Mićović, D Nežić, P Vuković, P Milojević, B Đukanović

**Affiliations:** 1 Cardiac Surgery, Cardiovascular Institute Dedinje, Belgrade, Serbia

## Background

Surgery for acute aortic dissection is always challenging, especially in the case of cerebral malperfusion. Question remains open, whether to perform only aortic repair or to reconstruct arch vessels if there flow is severely impaired by disease process.

## Case description

This is a case of acute aortic dissection with multiple tears, occluding innominate artery and causing brain and right hand malperfusion. Patient underwent successful emergent surgery in deep hypothermic circulatory arrest with antegrade cerebral protection for complete replacement of innominate artery and hemiarch. Complete innominate artery was replaced during cooling period on 22°C.

## Conclusion

There is still no consensus about arch vessel repair in the case of complicated aortic dissection. This technique is promising as it do not increase circulatory arrest time and it is safe and reproducible for patients with cerebral malperfusion.

